# The mechanism of high contents of oil and oleic acid revealed by transcriptomic and lipidomic analysis during embryogenesis in *Carya cathayensis* Sarg.

**DOI:** 10.1186/s12864-016-2434-7

**Published:** 2016-02-16

**Authors:** Jianqin Huang, Tong Zhang, Qixiang Zhang, Ming Chen, Zhengjia Wang, Bingsong Zheng, Guohua Xia, Xianyou Yang, Chunying Huang, Youjun Huang

**Affiliations:** Nurturing Station for the State Key Laboratory of Subtropical Silviculture, Zhejiang A & F University, Lin’an, Hangzhou, 311300 P. R. China; School of Life Science, Zhejiang University, Hangzhou, 310058 P. R. China

**Keywords:** *Carya cathayensis* Sarg, Embryogenesis, Hickory, Lipid biosynthesis, Transcriptome sequencing

## Abstract

**Background:**

Hickory (*Carya cathayensis* Sarg.) accumulates more than 70 % oil and 90 % unsaturated fatty acids with considerably high oleic acid in its mature embryo. The concurrent global trancriptomic and lipidomic analyses provided a framework for better understanding of glycerolipid biosynthesis and metabolism in the hickory nut.

**Results:**

The synthetical regulation of numerous leading lipid-related genes harmonized with the oil accumulation and fatty acid conversion in embryo development. The high level of *ACCase* correlated positively with fatty acids *de novo* synthesis, and the synergy of *DGAT2* and *PDAT* promoted the TAG assembly, and *oleosins*, *caleosins* and *steroleosins* were transcribed considerably high for timely energy reserve in oil body. Glycolysis possibly provided sufficient precursors and energy for lipid synthesis. The perfect harmonization of the high level of *SAD* with low level of *FAD2* facilitated the oleic acid accumulation. And the ratio of *FATA*/*FATB* or SAD/FATB was proposed for determining the saturated degree of oil. The gene multi-copy event was generated probably for accommodating various survival environments. A thermotolerant defense system including TAG hydrolysis determinants, heat shock proteins, and high ratio of MUFA to PUFA constrained the lipid degradation and provided a guarantee for high lipid content. A batch of potential genes recruited from the co-expression network helps us to understand the lipid synthesis and the response to high temperature better.

**Conclusions:**

The high transcriptional levels of key genes in lipid synthesis promoted the oil accumulation, and the harmonious expression of key ones for unsaturated fatty acids led oleic acid to high levels.

**Electronic supplementary material:**

The online version of this article (doi:10.1186/s12864-016-2434-7) contains supplementary material, which is available to authorized users.

## Background

Tree nuts are recommended as an important resource of healthy diet in human populations throughout the world, since they are rich sources of monounsaturated fatty acids (MUFAs), polyunsaturated fatty acids (PUFAs), however low in saturated fatty acids (SFAs) [1]. Recent scientific research has demonstrated that a high proportion of MUFAs or a high ratio of MUFAs to SFAs reduces cancer death and incidence [2]. Dietary linoleic acid (18:2, an n-6 fatty acid of PUFAs) intake reduces low-density lipoprotein cholesterol and risk of coronary heart disease, hypertension and type 2 diabetes [3]. A recommended minimum intake levels for essential fatty acids are 2.5 % linoleic acid plus o.5 % linolenic acid (18:3, an n-3 fatty acid of PUFAs) to prevent deficiency symptoms in adults, while the SFAs intake level keeps as low as possible [4].

Hickory (*Carya cathayensis* Sarg.) is one of the most productive woody oil-bearing plant in China. A mature hickory embryo contains more than 70 % oil and 90 % unsaturated fatty acids [5]. As a predominant composition of hickory oil, oleic acid (18:1) accounts for more than 70 % of the total unsaturated fatty acids content at maturation stage. Besides oleic acid, other compositions include a moderate amount of linoleic acid (~20 %) and low concentrations of palmitic (~4 %), stearic (~2 %), and linolenic acid (~5 %) [6].

To date, knowledge of oil accumulation in plants is based almost on studies of oil seeds. The skeletal pathway of fatty acid and lipid synthesis leading to triacylglycerol (TAG) production is basically understood. The fatty acid *de novo* synthesis occurs in plastid with hundreds of annotation genes in Arabidopsis [7, 8]. Acetyl-CoA, the substrate of fatty acid synthesis, is generated from pyruvate under catalysis by plastidial pyruvate dehydrogenase complex (PDHC) [9]. Subsequently, acetyl-CoA carboxylase (ACCase) converts acetyl-CoA to malonyl-CoA as the first rate-limiting step of fatty acid *de novo* synthesis [10]. Then, the carbon flux comes into reaction via fatty acid synthase (FAS), a multiple monofunctional enzymes complex. The series of enzymes catalyze acyl-ACP and result in the generation of two saturated acyl-ACPs, i.e., 16-carbon palmitoyl-ACP and 18-carbon stearoyl-ACP which are subsequently desaturated by delta-9-stearoyl-ACP desaturase (SAD). The resulting free long-chain fatty acids (16:0, 18:0, and 18:1) are esterified by long-chain acyl-CoA synthetase (LACS) and exported to endoplasmic reticulum for further participating acyl-editing by lysophosphatidylcholine acyltransferase (LPCAT) and phosphatidic acid phosphatase (PAP) [11, 12]. As the long-chain acyl-CoA and glycerol-3-phosphate (G3P) enter TAG assembly, the sequential reactions in so-called Kennedy pathway were performed by a series of enzymes [13, 14]. In the last step of Kennedy pathway, it is presumed that two isoforms of acyl-CoA: diacylglycerol acyltransferase (DGAT), the major enzymes catalyzing diacylglycerol (DAG) to produce TAG, play different roles in seed development of both Arabidopsis and oil crops. Moreover, several transcription factors such as WRINKLED1 (WRI1), PII, and two isoforms of LEAFY COTYLEDON (LEC1 and LEC2) play vital roles in plastidial glycolysis and pyruvate dehydrogenase action, and in lipid synthesis during seed maturation in Arabidopsis [15].

Nevertheless, the issue of high contents of oil and MUFA in tree nuts e.g., pecan, hickory, walnut and filbert was still not addressed. In this paper, dynamic morphological, physiological and biochemical characteristics, and transcriptome sequencing during embryo development in hickory in two sequential growing seasons and a complementary comparison of transcriptome with high-SFA oil palm were employed to discover the hickory’s high-oil and high-MUFA mechanism [16]. This work will give insight into the mechanism of oil deposition and fatty acids saturation conversion in the process of embryo development in oil seeds.

## Results and discussion

### Morphological and physiological-biochemical characteristics of developing embryo

**Fig. 1:**
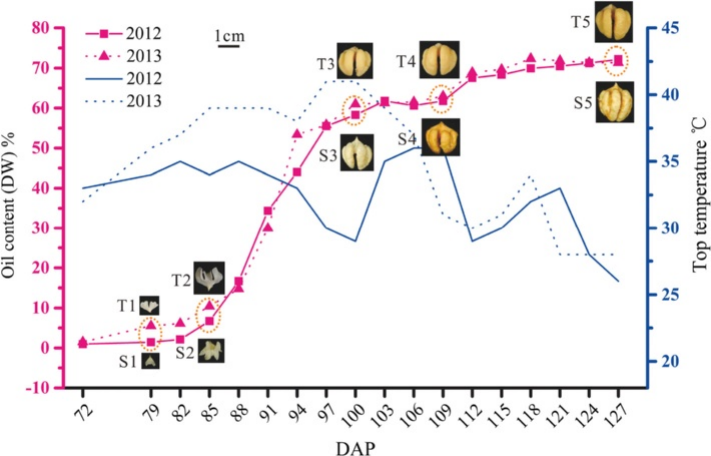
Morphological characteristics, oil accumulation and temperature variation in the process of hickory embryo development. The solid and the dashed red lines indicate the curves of oil content during the embryo development in 2012 and 2013, respectively. And, the solid and the dashed blue lines indicate the curves of top atmosphere temperature with the embryo development in two years, respectively. DAP indicates day after pollination. S1-S5 or T1-T5 indicate 5 temporal developmental stages, i.e., early cotyledon stage, midcotyledon stage, late cotyledon stage, full cotyledon stage, and maturation stage in 2012 and 2013 respectively, and chosen for RNA-seq analysis

The morphological, physiological and biochemical changes of developing embryo were tracked over the hickory seed development, and the trajectory of oil deposition in two successive growing seasons was depicted as Fig. 1. The embryos were developing from cotyledonary young ones on 72 DAP (day after pollination) to maturation on 127 DAP. The oil content was continuously accumulated from less than 2 to more than 70 % during the process. The dynamic oil accumulation was synchronous with the embryo development each year. According to the embryo dimension and its oil content, five developmental stages, i.e., early cotyledon stage, midcotyledon stage, late cotyledon stage, full cotyledon stage, and maturation stage, designated as S1-S5 in 2012 and T1-T5 in 2013 respectively, were chosen for RNA-seq analysis.

**Fig. 2:**
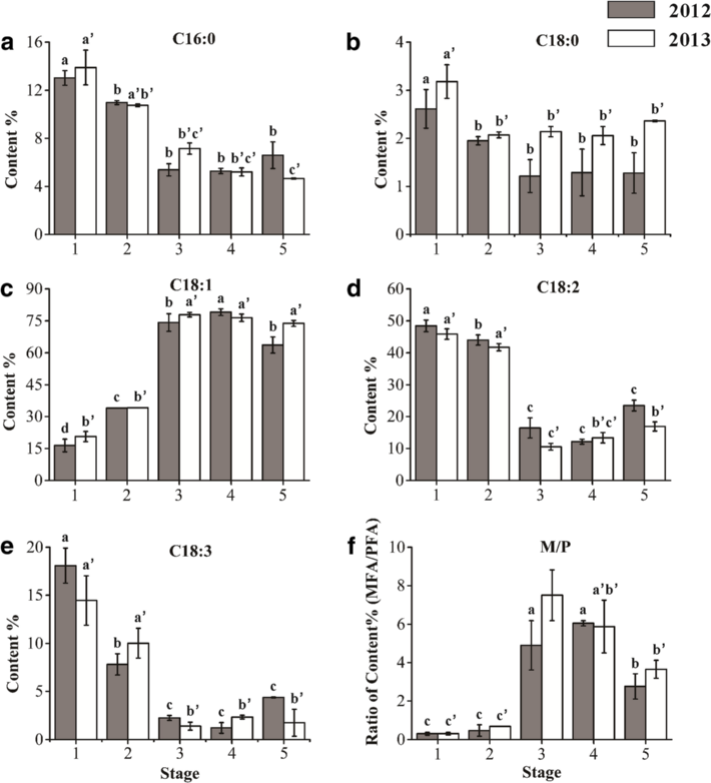
The contents or ratios of the main fatty acid composition. **A**-**E** indicate the contents of C16:0, C18:0, C18:1, C18:2 and C18:3 at 5 developmental stages each year. **F** indicates the dynamic ratio of MUFA to PUFA (M/P) at 5 stages in 2 years. Different lowercase letters denoted significant differences between different stages each year (*P* < 0.05)

Moreover, the fatty acid compositions of hickory oil at different stages were further investigated (Fig. 2). The contents of palmitic acid and stearic acid, which were two predominant compositions of SFAs, kept low levels (less than 20%) even dropped gradually over the embryo development. On the contrary, the unsaturated fatty acid contents were more than 80% in stage 1 and climbed softly up to approximately 93% of total fatty acids at stage 4 and 5. The oleic acid content was increasing from about 20 % at stage 1 to 80 % at stage 3 and keeping constant at stage 4–5. Rather, the mount of linoleic acid was high at stage 1–2 (approximately 40–50 %), but dropped rapidly down to roughly 10 % then kept low levels at the late stages. Analogously, linolenic acid reached relative high levels (average 18.08 % and 14.46 % each year, respectively) at stage 1, and declined to 10 % or 8 % at stage 2, then dropped rapidly down to single digits at stage 3–5. It was noted that the ratio of MUFAs/PUFAs at T3 was significantly higher than that at S3. Correspondingly, an excessive high temperature (41 °C) happened at T3 comparing to the normal S3 (29 °C). In soybean seedlings, the MUFA/PUFA ratio increased as a temperature shift from 25 to 35 °C [17]. And increased MUFAs could have contributed to the maintenance of membrane fluidity in legume seeds [18]. These conforming results suggested that the hickory embryo produced more MUFA and less PUFA to confine lipid oxidation under the hot wave. Taken together, a mature hickory nut contained more than 70% of oil, herein more than 90 % unsaturated fatty acids (including 63.65 % and 73.81 % oleic acid, 23.43 % and 16.90 % linoleic acid, 4.37 % and 1.74 % linolenic acid each year, respectively), less than 10 % SFAs.

### Transcriptome sequencing in sequential growing seasons

**Fig. 3:**
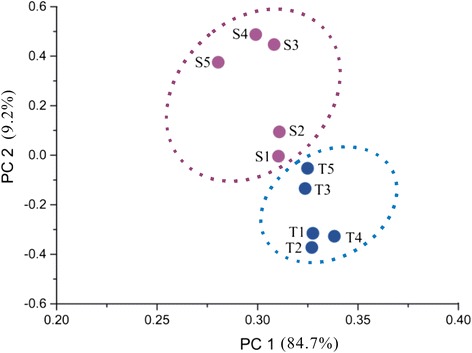
PCA plots of ten RNA-seq datasets normalized by log10 of FPKM in 2012 (S1-S5 in lilac) and 2013 (T1-T5 in dark blue). The plots showed that the first two principal components contained 84.7 % and 9.2 % variances, respectively

**Fig. 4:**
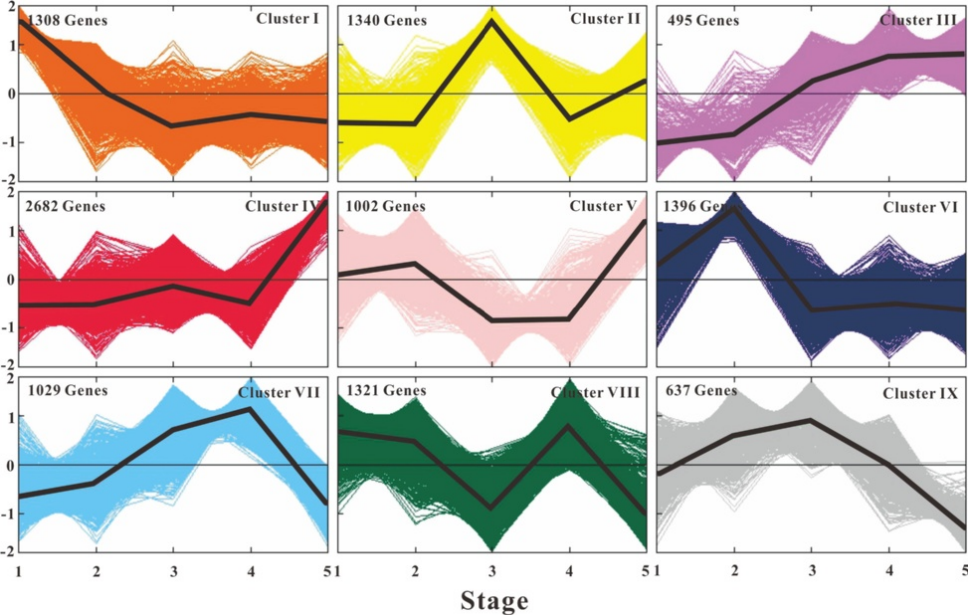
Dynamic expression patterns of differentially transcribed genes during embryo development. Nine major type patterns were identified, which were denoted as different color, respectively

**Fig. 5:**
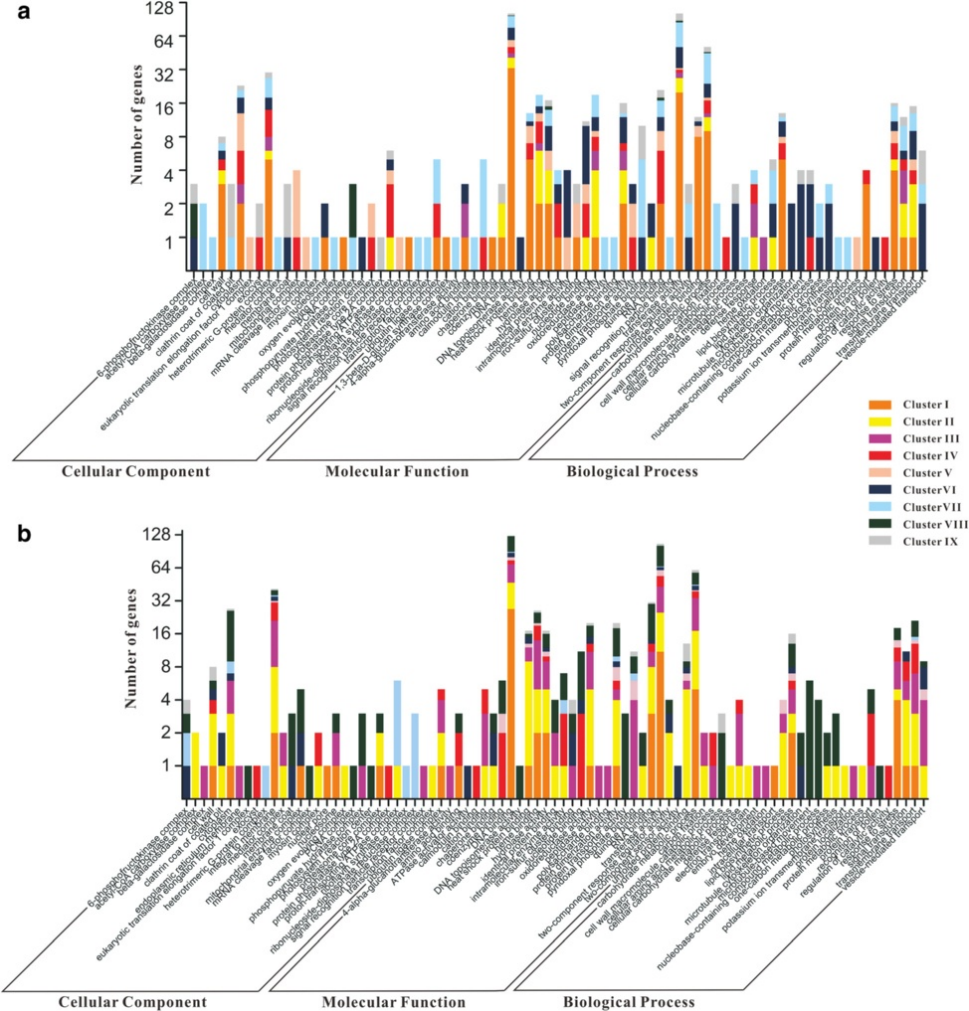
Functional categorization of differentially transcribed genes from different clusters. The color sets for each cluster are in strict accordance with Fig. 3. Panels **a** and **b** indicate the functional categorization in 2012 and 2013, respectively.

To dissect the molecular metabolism of oil accumulation over the embryo development in hickory, ten mRNA-seq databases (five databases each year, Fig. 1) were assembled into 86,432 contigs. And a total of 16,477 genes were annotated through BLASTN searches against a local *A. thaliana* cDNA sequence database. Principal component analysis (PCA) was implemented for consistency analysis between RNA-seq data of 2012 and 2013. The PCA plots showed that the first two principal components contained 84.7 % and 9.2 % variances, respectively. Apparently, the contribution of PC1 was much more than that of PC2. Hence, it sounded reasonable that the ten RNA-seq datasets were well clustered (Fig. 3). And the annual RNA-seq datasets were also well-clustered, although S5 and T4 were somewhat deviated from other datasets each year. In order to analyze the gene expression pattern of the whole sequencing data, a total of 5,606 differentially transcribed genes were clustered into 9 major patterns (Fig. 4). Furthermore, the differential-transcribed genes reflecting to the Gene Ontology at level 4 were classified into three functional groups, i.e., cellular component, molecular function and biological process (Fig. 5). Several functional groups related closely with lipid synthesis such as acetyl-CoA carboxylase complex, lipid biosynthetic process, and lipid metabolic process were enriched. For instance, the functional group of acetyl-CoA carboxylase complex belonged to Cluster VII in 2012 and Cluster II in 2013, suggesting that the transcriptional level of *ACCase* rose up to a peak at S4 and T3, respectively. The functional group of lipid biosynthetic process belonged discretely to Cluster II, VI, VII and IX in 2012, but mainly to Cluster II in 2013. The fact hinted that the genes related to lipid synthesis were strongly transcribed at T3 for facilitating oil accumulation under the hot wave in 2013. Furthermore, heat shock proteins (HSPs) functioned in several patterns in 2012 but functioned mainly in cluster II pattern in 2013 whose genes were transcribed higher to a peak at T2-T3 then did lower afterwards, suggesting that the thermotolerance of HSPs alleviated proudly the injury of the hot wave [19] .

### Dynamic differential transcription in lipid synthesis

**Fig. 6:**
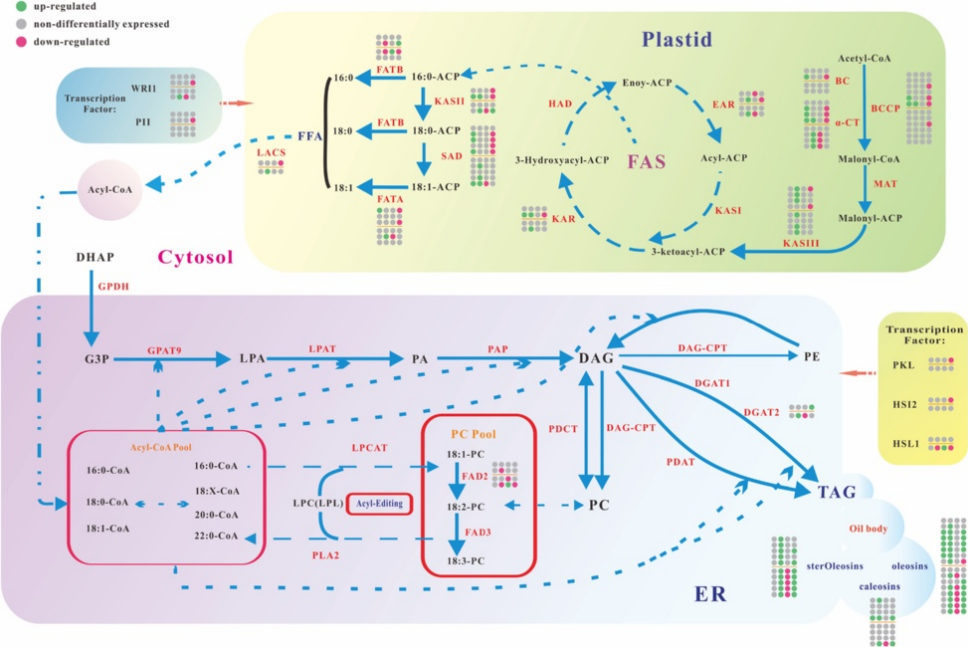
The differential transcription of lipid synthesis genes. The blue solid dot indicates the up-regulated gene repeat, while the red solid dot indicates the down-regulated gene repeat. The gray dot indicates the non-differentially expressed gene repeat. The upper and lower parts of each gene indicate the dynamic expression in 2012 and 2013, respectively

The dynamic differential transcription of genes related with fatty acid *de novo* synthesis, TAG assembly and reserve in two years was studied (Fig. 6; Additional file 1). In detail, the three subunits of *ACCase* (*BC*, *α-CT* and *BCCP*), *3-ketoacyl-ACP reductase* (*KAR*), *ketoacyl synthase II* (*KASII*), *SAD*, *FATA* and *oleosins* were up-regulated, while none was down-regulated at S2 stage, suggesting that the fatty acid synthesis initiated accelerating and storing the target products in oil bodies. Simultaneously, the oil content rose from 1.48 to 6.74 % during stage 1–2. Moreover, the up-regulation of *SAD* and *FATA* provided a hint that unsaturated fatty acids became predominant constituents in the anabolites. At S3 stage, two *ACCase* subunits (*α-CT* and *BCCP*), *KASIII*, *KAR*, *enoyl-ACP reductase* (*EAR*), *KASII*, *LACS*, *steroleosins*, *caleosins* and *oleosins* were up-regulated, while *FATB* and ω-3 fatty acid desaturase 2 (*FAD2*) were synchronously down-regulated. The fact demonstrated that the fatty acids were more rapidly synthesized and their corresponding lipids were instantly transferred to oil bodies for energy reserve. The down-regulation of *FATB* suggested that the unsaturated fatty acids rather than saturated ones was massively produced at this stage. The down-regulation of *FAD2* restrained the PUFAs output but promoted simultaneously the MUFAs output. Meanwhile, the oil content rose sharply to 50.30 %, and the content of predominant component C18:1 in oil reached 74.22 %. The contents of C18:2, C16:0 and C18:3 were 16.43 %, 5.38 % and 2.23 %, respectively. On the whole, the contents and constituents of total lipids matched well with transcriptional levels of differentially-expressed genes which were associated with lipid synthesis. At S4 stage, *EAR* was down-regulated, but other lipid-related genes did not meet the threshold of differential transcription. Whatever, the slowdown of fatty acid synthesis caused a minor increase of oil content which rose no more than 3 percentage points to 61.78 % with an elevated proportion (79.11 %) of C18:1, a declined proportion (12.14 %) of C18:2, and a constant proportion of other lipid components. At the last stage, the down-regulation of numerous genes involving in fatty acid *de novo* synthesis such as *ACCase*, *MAT*, *KASIII*, *KAR*, *EAR*, *KASII*, *SAD*, *FATA*, and *LACS* led to a sharp decrease of fatty acid synthesis. Nevertheless, several genes which dominated TAG assembly or its storage, such as *diacylglycerol acyltransferase* (*DGAT2*), *steroleosins*, *caleosins*, and *oleosins*, were up-regulated, indicating that TAGs were accumulated and stored rapidly in oil bodies at maturity. As a result, the oil content reached 72.15 % finally. Interestingly, C18:1 made a significant decrease (from 79.11 to 63.65 %), while C18:2 did a distinct increase (from 12.14 to 23.43 %). As we know, FAD2 is a specific enzyme which catalyzes C18:1 to C18:2 [20]. The differentially-transcribed *FAD2* kept a relatively high level at stage 1–2, but declined sharply at the following stages. The dynamic transcription pattern of *FAD2* led linoleic acid and linolenic acid to high levels at early stages and low levels at later stages. However, no *FAD3* homolog could be identified from the two-year transcriptomic data. It was contradicted with the proportional linolenic acid in the process of embryo development. Luckily, the discovery of plastidic FAD8 in hickory smoothed the conflict over. In birch leaves, low temperature induced the expression of *BpFAD3* and *BpFAD8* and a synchronous increase of 18:3 occurred in TAG [21]. Elevated temperatures decreased the expressional level of FAD8 in Arabidopsis [22, 23]. Over-expression of FAD8 imposed much greater heat sensitivity than does FAD3 overexpression in Arabidopsis and tobacco [24, 25]. In hickory, the expressional levels of *FAD8* at T2-T3 were remarkably lower than those at S2-S3, suggesting that *FAD8* compromised with the hot wave in 2013. Consequently, it was presumed that the linolenic acid in the developing hickory embryo was predominately generated by *FAD8* in plastid rather than *FAD3* in endoplasmic reticulum.

Similarily, the differential transcription of these lipid-related genes in the subsequent growing season was detailedly described as Fig. 6. Through a comparison of two-year data, the discrepancies of differential transcription of lipid-related genes between the two growing seasons were further explored as follows. At stage 4, for instance, only *EAR* was down-regulated in 2012, while a number of genes besides *EAR*, such as *α-CT*, *KASII*, *SAD*, *FATA*, *DGAT2*, *steroleosins*, *caleosins*, and *oleosins*, were down-regulated in 2013. Furthermore, it was noted that C18:2 content had a minor increase at T4, while it did not happen at S4 yet. In addition, as the atmosphere temperature at S3-S4 stages kept constant, no gene was differentially-transcribed with an exception of *EAR* up-regulation. However, as the atmosphere temperature at T4 stage fell down from 36 to 29.7 °C, numerous genes for fatty acid synthesis, *DGAT2* for TAG assembly, and three genes (i.e., *steroleosins*, *caleosins*, and *oleosins*) related with energy reserve in oil bodies were down-regulated. Together, the above-mentioned fact elucidated that the hot wave impaired little to the enzymes activities in lipid biosynthesis, while the dropping temperature could restrain their activities. It suggested that there lived a certain protective mechanism in hickory to accommodate the hot wave, which attracted us making a specific discussion below for addressing this issue.

Furthermore, several crucial transcription factors were differentially transcribed over the embryo development in the two sequential growing seasons. For instance, the transcriptional level of *WRI1* was peaked at S3, reduced at S4, and down-regulated significantly at S5; while its transcription was up-regulated at T3 and down-regulated significantly at T4 stage. Similarly, the transcriptional levels of another transcription factor *PII* went up at early 3 stages then declined afterwards in each growing season. Other several transcription factors such as *HSI2*, *HSL1* and *PKL* were differentially transcribed at different stages. However, the transcription of *LEC2* and *FUS3* did not reach the threshold of significant difference over the entire embryo development in the two growing seasons.

### *ACCase* transcription correlated positively with fatty acid *de novo* synthesis

**Fig. 7:**
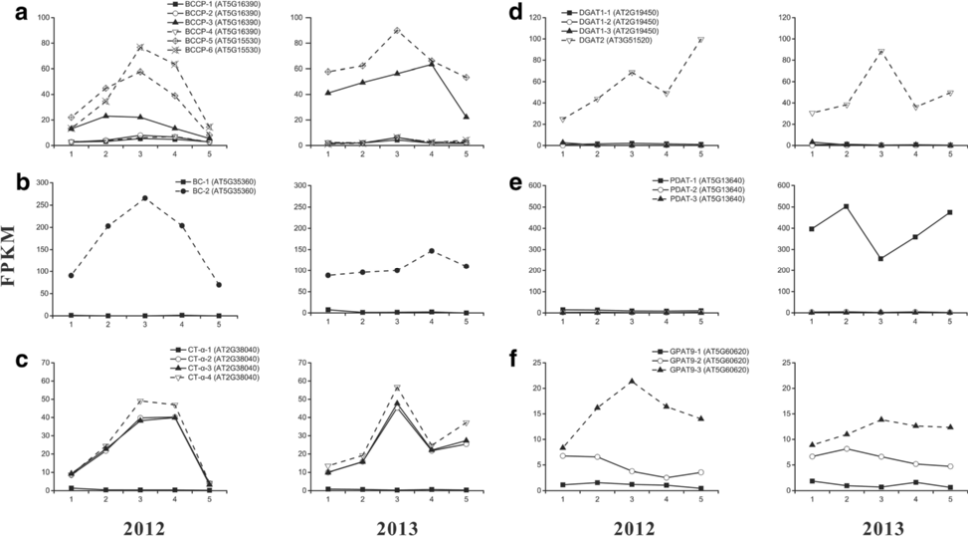
Temporal changes in transcriptional levels for skeletal lipid synthesis enzymes during embryo development in 2 years. **a**, **b** and **c** indicate the dynamic transcription of the 3 subunits (*BC*, *α-CT* and *BCCP*) of *ACCase* in 2012 and 2013, respectively. And, **d**, **e** and **f** indicate the dynamic transcription of *DGAT1*, *DGAT2*, *PDAT* and *GPAT9* in two years, respectively. All repeats of each gene are respectively shown in the panel

Plant ACCase acts as a key control point over the flux of carbon into fatty acids. The catalysis efficient of ACCase was determinant for transfer from photosynthate to lipid [15]. The dynamic transcription of *ACCase* subunits over the hickory embryo development in the two growing seasons was depicted in Fig. 7. Of 6 *BCCP* repeats, *BCCP-3* (AT5G16390), *BCCP-5* (AT5G15530) and *BCCP-6* (AT5G15530) became dominant ones because their transcriptional levels rose at early stages and reached a peak at S3 stage, then fell down afterwards in first growing season. In regard to the second growing season, *BCCP-6* transcription kept a very low level, while the transcriptional levels of *BCCP-3* and *BCCP-5* were higher than those in first growing season (Fig. 7a). The result implied the different contributions of these *BCCP* repeats for fatty acid *de novo* synthesis. Considering the hot wave in 2013, the fact of the lower transcriptional level in 2013 and higher level in 2012 of *BCCP-6* could attribute to temperature variation. That is, this *BCCP* repeat seemed a gene vulnerable to temperature disturbance, and the hot wave confined its transcription. Conversely, *BCCP-3* and *BCCP-5*, particularly the latter, transcribed higher under the hot wave, suggesting that both *BCCP* repeats accommodated the elevated temperature to ensure the normal lipid biosynthesis. Of 2 BC repeats, *BC-1* (AT5G35360) transcribed at very low levels (FPKM (fragments per kilobase of exon per million fragments mapped) < 10) over embryo development in 2 years; while *BC-2* (AT5G35360) transcribed at relative high levels, whose FPKM ranged 69.67–265.36 in first growing season and ranged 88.50–147.01 in second growing season (Fig. 7b). It was indicated that the *BC-2* played an important role for the *ACCase* activity. Among 4 repeats of *α-CT* subunit (AT2G38040), 3 repeats (i.e., *α-CT-2*, *α-CT-3* and *α-CT-4*) showed a similar transcription pattern which was up-regulated continuously at stage 1–3 but down-regulated subsequently, while the transcription of the rest repeat (*α-CT-1*) kept low all the time (Fig. 7c). The expression pattern provided a reasonable explanation for the rapid oil accumulation at third stage each year.

### *DGAT2* and *PDAT* played crucial roles in TAG assembly

As a limited enzyme, DGAT catalyzed the last step of TAG synthesis [26]. In hickory, the transcription of all the three repeats of *DGAT1* (AT2G19450) kept at a low level all along, while the sole *DGAT2* (AT3G51520) repeat transcribed differentially in a wide range in both growing seasons (Fig. 7d). Concretely, *DGAT2* was up-regulated continuously at stage 1–3 and down-regulated at stage 4 then up-regulated at stage 5. The result certified the vital role of *DGAT2* rather than *DGAT1* in TAG assembly in hickory.

Phospholipid:diacylglycerol acyltransferase (PDAT) implemented the direct transfer of a fatty acid from phosphatidylcholine to DAG producing TAG [14]. In first year, all the three repeats of *PDAT* kept little transcribing throughout the entire embryo development. In second year, 2 (*PDAT-2* and *PDAT-3*) of the three repeats (AT5G13640) still kept a subtle transcription all along (Fig. 7e). However, the remaining repeat, i.e., *PDAT-1*, was high transcribed over the embryo development. Noteworthily, as *DGAT2* was high transcribed, *PDAT* was low done synchronously, vice versa. The result demonstrated that the complementation of *PDAT* and *DGAT2* impelled the TAG assembly. It was further interpreted that *DGAT2* was responsible for catalyzing the last committing step of TAG synthesis, while *PDAT* kept silent as a reserve at moderate temperature. In case of unfavorable condition such as the hot wave in summer, *PDAT* and *DGAT2* on behalf of phosphatidylcholine pathway and Kennedy pathway respectively were triggered for ensuring the unaffected TAG synthesis. In Arabidopsis, PDAT and DGAT1 are the major enzymes with overlapping functions for catalyzing TAG production [27, 28]. *DGAT2* function remained unclear in Arabidopsis. In some plants such as *Ricinus communis* and *Vernicia fordii*, *DGAT2* homologs were more highly expressed than *DGAT1* ones during seed maturation [29, 30]. In *R. communis* and *Aleurites montana*, *DGAT2* was proposed to be important for incorporation of unusual fatty acids into TAG [31]. *DGAT2* was also abundant in olive [32] and oil palm [16, 33] for accumulating normal TAG. Our study certified the roles of *PDAT* and *DGAT2* and their overlapping functions in TAG synthesis in different environments in hickory.

### High transcription of *SAD* and *FAD2* were associated with oleic acid production

**Fig. 8:**
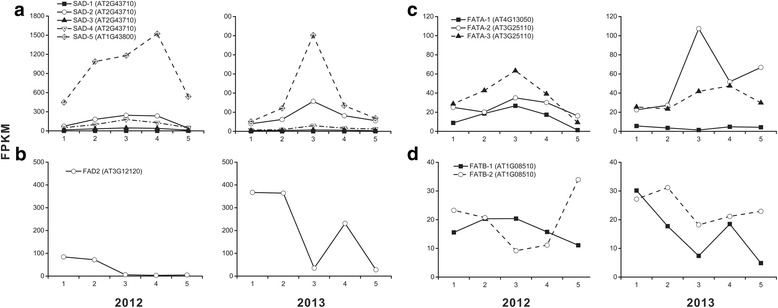
Temporal changes in transcriptional levels for unsaturated fatty acid synthesis enzymes (SAD, FAD2 and FATA) and saturated fatty acid synthesis enzyme (FATB) during embryo development in 2 years. Panels **a**, **b**, **c** and **d** indicate the dynamic expression of *SAD*,* FAD2*, *FATA* and *FATB*, respectively.

SAD plays a crucial role for *de novo* synthesis of unsaturated fatty acids in plants [34]. Among 5 repeats of *SAD* in hickory, *SAD-5* (AT1G43800) was transcribed remarkably, followed by *SAD-2* (AT2G43710). The up-regulation of both repeats gave rise to produce abundant unsaturated fatty acids during S1-S4 or T1-T3 (Fig. 8a). *FAD2* encodes a ω-6 desaturase that converts oleic acid to linoleic acid [34]. In hickory, the fact of the high level at the first two stages and low level at later stages of *FAD2* demonstrated a dominant production of PUFAs in early embryo development and a major production of MUFAs in late embryo development. Strikingly, the expression of *SAD* was contrary to that of *FAD2* during stage 1–4 (Fig. 8b), suggesting that SAD catalyzed rapidly the synthesis of unsaturated fatty acids in the upstream of lipid biosynthesis, while FADs controlled the ratio of MUFAs to PUFAs in the downstream. Consequently, the perfect harmonization of both enzymes’ expression facilitated the high content of oleic acid.

In Arabidopsis, SAD transcription was more abundant than any other enzymes involved in fatty acid synthesis [35]. Of 7 SAD isoforms, At2g43710 is the most highly expressed in Arabidopsis seeds. Of note, orthologs of At2g43710 were also the most abundant in developing seeds of *R. communis*, *Brassica napus*, *Euonymus alatus* and *Tropaeolum majus* [35]. However, the gene encoding the SAD isoform in hickory with the highest transcription was AT1G43800 rather than At2g43710, implying a specific mechanism of fatty acid desaturation in this species.

### Comparison of high-lipid and high-oleate mechanism in oil plants

To gain insight into the high-lipid mechanism of in hickory, a comparison was implemented among transcriptome databases of lipid synthesis in hickory, oil palm and date palm which were derived from the open access (http://www.pnas.org/lookup/suppl/doi:10.1073/pnas.1106502108/-/DCSupplemental/sd02.xls). According to the literature [16], oil palm accumulates up to 90 % oil in its mesocarp, while the closely related date palm accumulates almost exclusively sugars instead of oil. First, the transcript of *ACCase* was abundant in hickory and oil palm, but little in date palm. The expressional levels of the three subunits of *ACCase* in hickory, i.e., *α- CT*, *BCCP* and *BC*, displayed a coordinated temporal pattern which went up to a peak at stage 3–4 and decline during late embryo maturation (Fig. 7c). The three *ACCase* subunits in oil palm were transcribed much higher than those in date palm during fruit development. Also, the *DGAT* homologs in hickory were transcribed actively during embryo development in two growing seasons (Fig. 7d), so did the homologs in oil palm. However, their paralogs of *DGAT* transcribed less in date palm mesocarp. Besides the two genes coding for committing enzymes which determined the synthetic direction and rate of the first step and the last step of lipid synthesis, other kernel genes coding for main enzymes in fatty acid *de novo* synthesis such as *KASIII*, *KAR*, *hydroxyacyl-ACP dehydratase* (*HAD*), *EAR*, and *KASII* were transcribed as a coordinated pattern similar to *ACCase* in the three oil trees. Interestingly, almost all important enzymes in Kennedy pathway for TAG assembly such as *glyceraldehyde-3-phosphate dehydrogenase* (*GPDH*), *glycerol-3-phosphate acyl-transferase* (*GPAT9*), *lysophosphatidic acid acyl transferase* (*LPAT*), *PDAT*, and *DGAT* were transcribed at low levels. The transcriptional levels of the first four enzymes in date palm seemed not significantly lower than those in oil palm. It was suggested that oil accumulation relied on the efficiency of fatty acid *de novo* synthesis rather than that of TAG assembly except the last committed step of converting diacylglycerol (DAG) to TAG catalyzed by DGAT. Three genes in oil body, i.e., *oleosins*, *caleosins* and *steroleosins* were transcribed considerably high in hickory than those in oil palm and date palm, suggesting that there lied a specific oil storage mechanism in hickory.

**Fig. 9:**
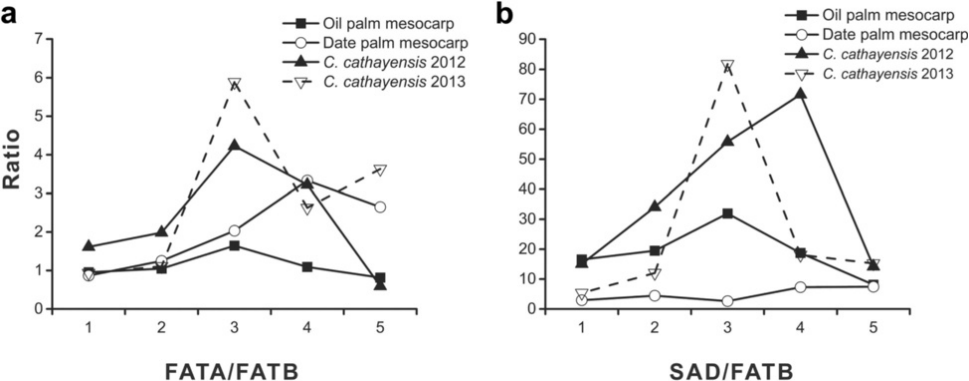
Temporal changes in index of saturated degree during embryo development in 2 years. The value of FATA/FATB and SAD/FATB correlated negatively with the saturated degree. Panels **a** and **b** indicate the dynamic value of FATA/FATB and SAD/FATB, respectively.

Furthermore, the transcriptional level of *FATA* was generally higher than that of *FATB*, which led to a greater production of unsaturated fatty acids than that of saturated ones in several plants seeds [36]. In *R. communis*, the transcriptional level of *FATA* were 1000-fold higher than that of *FATB*. The ratio of FATA to FATB provided an evidence for the very low content of SFAs (~2 %) in *R. communis* seeds [37]. The ratios of *FATA*/*FATB* and *SAD*/*FATB* in these plants were further calculated for unsaturation analysis (Fig. 9a, b). Oil palm and date palm contained high content of saturated palmitic acid (approximately 40% of total fatty acids) [16] and low *FATA*/*FATB* and *SAD*/*FATB* ratios. These low ratios led both palms to a low proportion of unsaturated fatty acids and a high proportion of SFAs. On the contrary, the ratios in hickory embryo reached maximums at stage 3–4 in the successive growing seasons, suggesting the rapid synthesis of unsaturated fatty acids in this duration. The fact illustrated the high unsaturation (more than 90% of unsaturated fatty acids) of oil in hickory. Together, the ratio of *FATA*/*FATB* (or *SAD*/*FATB*) could be applied for determining the saturated degree of oil.

### Glycolysis possibly provided precursors and energy for lipid synthesis

**Fig. 10:**
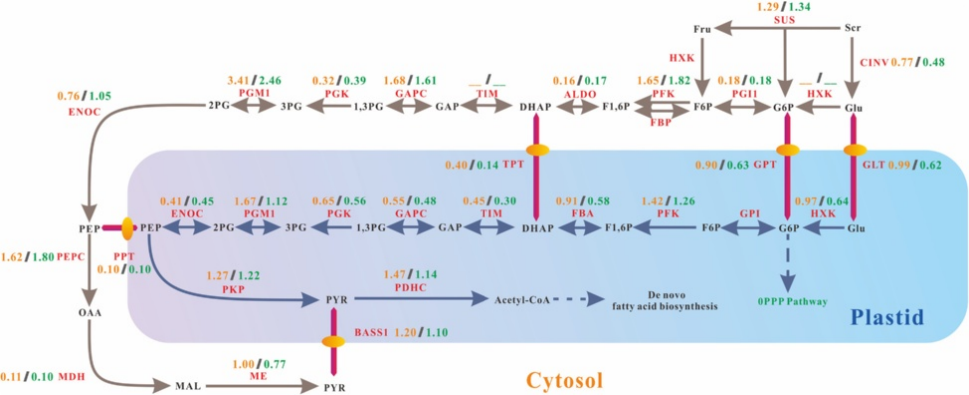
The differential transcription of glycolysis genes. The two-way arrow indicates a reversible reaction, while the one-way arrow indicates an irreversible reaction. The solid circle in yellow indicates a translocator. The value in brown indicates the ratio of total FPKM in 2013 to that in 2012, while the green value indicates the fold change of FPKM at stage 3 in 2013 to that in 2012

Glycolysis converted sugar to numerous precursors for protein and fatty acid synthesis concomitantly producing ATP by substrate level phosphorylation [38]. About 90 % of glucose fed to developing canola embryo was converted to pyruvate by glycolysis [39]. In hickory, the consistency of differential transcription profile between glycolysis and lipid synthesis was generally well confirmed (Fig. 10).

**Fig. 11:**
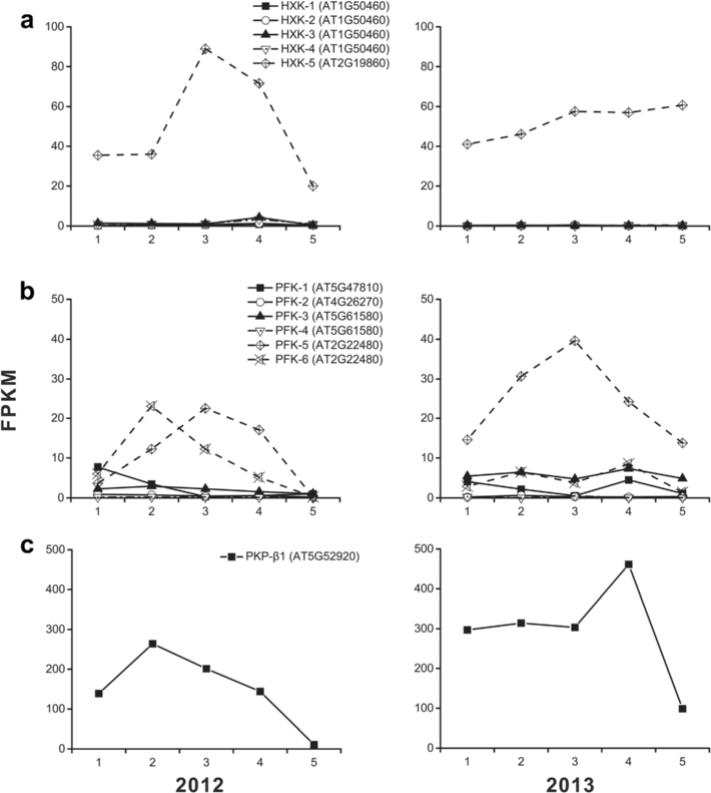
Temporal changes in transcriptional levels for committing enzymes (HXK, PFK, and PKP-β1) of glycolysis during embryo development in 2 years. Panels **a**, **b** and **c** indicate the dynamic expression of HXK, PFK and PKP-β1, respectively.

The conversion of glucose to glucose-6-phosphate (G6P) catalyzed by hexokinase (HXK) was the first and committing step in glycolysis [40]. A *HXK* homolog in hickory designated as *HXK-5* (AT2G19860) was intensively up-regulated at S3 and S4 and sharply down-regulated afterwards in first year, while the transcriptional level of the homolog climbed to T3 and kept the height at later stages in following year (Fig. 11a).

Phosphofructokinase (PFK) was the other limited enzyme which catalyzed G6P to produce glucose 1,6-bisphosphate (G1,6P) [41]. Of 6 *PFK* repeats, *PFK-5* (AT2G22480) and *PFK-6* (AT2G22480) were high transcribed in first year. However in second year, merely *PFK-5* played a leading role, whose transcriptional level went up and reached a peak then fell down (Fig. 11b). Interestingly, the sum of FPKM of *PFK-5* and *PFK-6* in each stage in first year was almost equivalent to that in second year. It led us to predict that the enzyme encoded by *PFK-5* was of the better tolerance to the hot wave than other isozymes.

The third well-known limiting step was the biochemical reaction from phosphoenolpyruvate (PEP) to pyruvate catalyzed by phosphoenolpyruvate (PKP). The PKP activity was determined by β1 subunit. Disrupting the PKP-β1 encoding gene caused a reduction of PKP activity and seed oil content [38]. In hickory, the *PKP-β1* homolog (AT5G52920) was transcribed relatively high at stage 1–4 and low at the last stage (Fig. 11c), suggesting that sufficient pyruvate was produced for meeting the demand of fatty acid *de novo* synthesis. Noting that the *PKP* FPKM at each stage in second year was higher than that in first year, the glycolysis was possibly impelled for producing more ATP and NADPH to relieve excessive energy consumption causing by the hot wave.

### Constraint of lipid degradation became a guarantee for high lipid content

**Fig. 12:**
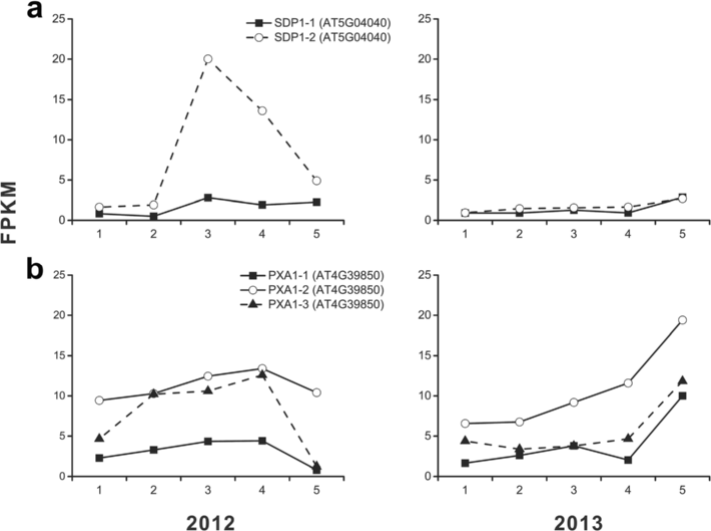
Temporal changes in transcriptional levels for committing enzymes (SDP1 and PXA1) of lipid hydrolysis during embryo development in 2 years. Panels **a** and **b** indicate the dynamic expression of SDP1 and PXA1, respectively.

The oil content is governed by the dynamic balance between synthesis and breakdown and a deficiency in TAG hydrolysis has been shown to cause an increase in oil deposition [42]. The first hydrolytic attack on the TAG molecule in oilseeds is primarily catalyzed by SUGAR-DEPENDENT1 (SDP1), an oil body-associated protein which hydrolyzes TAG to free fatty acids and DAG [42–45]. Suppression of the SDP1 during seed development enhances oil yield in *B. napus* [46]. Moreover, PEROXISOMAL ABC-TRANSPORTER1 (PXA1) is the other important protein that transported a variety of substrates into peroxisomes for their subsequent metabolism by β-oxidation in Arabidopsis [47]. Due to their determinant roles in TAG breakdown, the isoforms of *SDP1* and *PXA1* were studied for understanding the TAG hydrolysis over the hickory embryo development. Consequently, two *SDP1* repeats and three *PXA1* ones were uncovered from the transcriptome data. The transcription of both *SDP1* repeats kept at relatively low levels whose FPKM value in two years was not more than 20 (Fig. 12a). Furthermore, the three *PXA1* repeats in hickory were also transcribed at low levels (FPKM < 13) (Fig. 12b). Hence, the constraint of lipid degradation became a guarantee for high lipid content.

### Influence of hot wave on gene expression in lipid synthesis

The curve of the oil content in 2012 coincided with that in 2013, showing little influence of the hot wave on the oil accumulation (Fig. 1). The fact suggested that there lived a certain protective mechanism during embryo development to eliminate negative effects caused by the hot wave. As described above, the hot wave restrained heavily the transcription of *ACCase* subunits *BCCP* (*BCCP-6*) and *BC* (*BC-2*), and a *GPAT9* repeat (*GPAT9-3*); while it accelerated the transcription of other repeats of *ACCase* subunits *BCCP* (*BCCP-5* and *BCCP-3*), a *PDAT* repeat (*PDAT-1*), and a *FATA* repeat (*FATA-2*). These genes attempted to accommodate temperature variation through selective expression of different repeats for normal oil accumulation, suggesting that the multi-copy event in higher plants was required for adapting various survival environments.

**Fig. 13:**
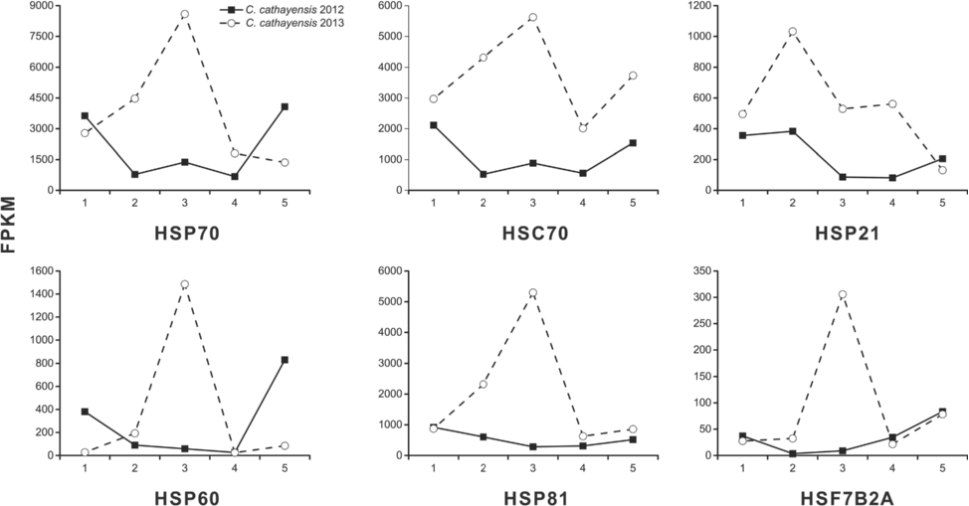
Temporal changes in transcriptional levels for heat shock proteins during embryo development in 2 years

Besides the lipid-related genes, other differential-transcribed genes against the hot wave during embryo development were also explored. Of them, the significantly up-regulated genes involved in ABA signaling pathway, jasmonic acid biosynthesis, HSPs, phospholipid signaling, sphingolipid biosynthesis, suberin synthesis and transport, and numerous transcription factors (*MYB*, *ABI5*, *NF-YC*, *BES1*, *DREB26*, *bHLH144*, *bHLH96*, *EGL3*, *ARF6*, *ARF1*, *NF-YA7*, *BIM2*, *ATWHY2*, *HAP2C*, *BZR1*, *ATRL6*, etc.). For example, almost all the heat shock proteins expressed much higher in 2013 than in 2012, particularly at stage 3 (Fig. 13). As one of the heat shock transcription factors, the *HSFB2A* was transcribed the highest at T3, suggesting its crucial role for high temperature tolerance [48]. Together, the high transcriptional levels of genes related to lipid synthesis and thermotolerance and the low transcriptional levels of genes related to lipid degradation protected hickory normal embryo development and oil accumulation against the hot wave.

### Potential genes related with lipid synthesis via co-expression network

**Fig. 14:**
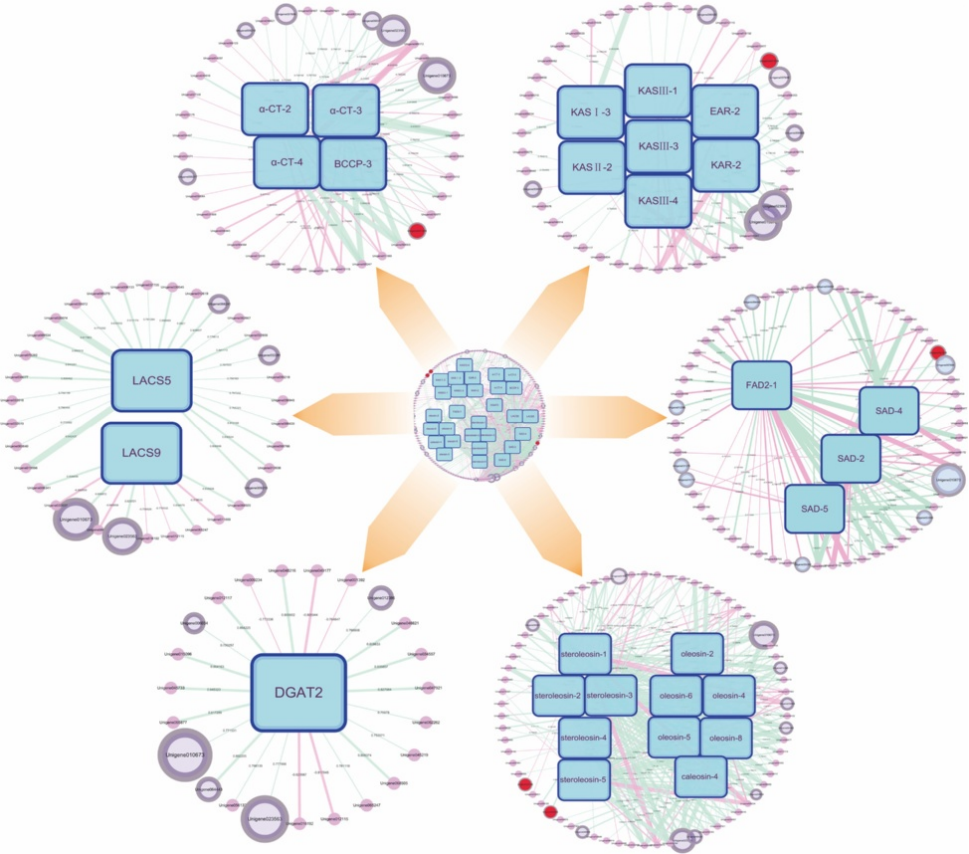
Model of gene co-expression network in hickory embryo development. The line width indicates the correlation strength between 2 genes. The red line indicates the positive correlation, while the green line indicates the negative correlation. The gene in rectangle indicates lipid-related one. The unigene in magnified circle indicates the co-expressed candidate gene. The unigene in red solid circle indicates a putative transcription factor

In order to identify potential novel genes associated with the lipid biosynthesis in hickory, a co-expression network was constructed from a genome-wide co-expresser search for lipid-related genes. The final network encompassed 844 unigenes co-expressed strongly with 29 lipid-related genes (PCC ≥ 0.7). Herein, 152 unigenes which homologs in other species were registered in NCBI-NR database were recruited as the potential genes, while the remaining uncharacterized ones in this database were abandoned for the time being due to the difficulty to further research (Fig. 14). Of the 152 unigenes with known function, several unigenes were associated directly with lipid biosynthesis, including two oleosin unigenes (i.e., Unigene012386 and Unigene023563), one unigene coding for acyl-carrier protein (i.e., Unigene008858), one uninene for phosphatidylinositol transfer protein (i.e., Unigene032291), and so on (Additional file 2). In addition, the other known unigenes contained one unigene coding for BP8 related with embryogenesis (Unigene009218); several unigenes related with resistance to stress, such as Unigene065941 coding for HSP, Unigene009019 and Unigene066123 coding for embryo dehydrins; Unigene022838, Unigene068241 and Unigene013012 coding for ascorbate oxidase precursor, polyphenol oxidase, and chaperone for superoxide dismutase, respectively; Unigene066520 coding for ethylene insensitive 3 for resistance to biotic and abiotic stress; Unigene053287 coding for NSP-interacting kinase 3 (a transducer of plant defense signaling); Unigene026336 coding for kelch repeat protein for oxidative stress, etc. And several transcription factors such as *ICE1* and *bHLH93* were uncovered. Moreover, several glycolytic genes coding for sugar phosphate translocator, hexose transporter, etc. were also discovered. For instance, Unigene065941 co-expressed strongly (PCC = 0.9079) with Unigene023573 which encoded an oil body protein namely caleosin. Through a local blast against the NR database, Unigene065941 was known as a heat shock protein in *R. communis*. It was inferred that some genes like Unigene065941 in hickory were triggered to accommodate various unfavorable environments including the hot wave to ensure the normal oil accumulation and energy reserve in oil bodies. Furthermore, both oleosin unigenes (i.e., Unigene012386 and Unigene023563) co-expressed strongly (PCC > 0.9) with the transcription factor *WRI1*. Unigene008858 coding for acyl carrier protein 1 also presented a significant co-expression with the *SAD* homolog. And the discovery of several glycolytic genes co-expressing with lipid-related genes suggested the close relationship between glycolysis and lipid biosynthesis.

## Conclusion

The two-year concurrent global trancriptomic and lipidomic analyses conducted in this study provide a framework for better understanding of glycerolipid biosynthesis and metabolism in the oleaginous nut of hickory (*Carya cathayensis* Sarg.). The synthetical regulation of numerous leading lipid-related genes harmonized with the oil accumulation and fatty acid constituent conversion in hickory embryo development. The high transcriptional level of *ACCase* correlated positively with fatty acid *de novo* synthesis, the synergy of *DGAT2* and *PDAT* promoted the TAG assembly, and *oleosins*, *caleosins* and *steroleosins* were transcribed considerably high for timely energy reserve in oil body. The oil accumulation in developing hickory embryo relied prevailingly on the efficiency of fatty acid *de novo* synthesis instead of that of TAG assembly except the last committed step of converting DAG to TAG. The perfect harmonization of the high level of *SAD* with low level of *FAD2* facilitated the high content of oleic acid in hickory. And the ratio of *FATA*/*FATB* or *SAD*/*FATB* was proposed for determining the saturated degree of oil in plants. Simultaneously, glycolysis possibly provided sufficient precursors concomitantly producing ATP and NADPH for lipid synthesis. The gene multi-copy event was generated probably for accommodating various survival environments. A thermotolerant defense system including TAG hydrolysis determinants of *SDP1* and *PXA1*, heat shock proteins, transcription factors, and high ratio of MUFA to PUFA constrained the lipid degradation and provided a guarantee for high lipid content. Moreover, a batch of potential lipid-related or thermo-inducing genes recruited from the genome-wide co-expression network helps us to understand the lipid synthesis and the response to high temperature better.

## Methods

### Plant material

The two-year hickory (No. of the superior tree is *C. cathayensis*-Linan-Taihuyuan-012) nuts were sampled from a 22-year-old hickory tree in Lin’an (30°N, 119°W), China in the process of seed development from the middle July to the early September in 2012 and 2013. The embryos were developing from cotyledonary young ones on 72^nd^ DAP to maturation on 127 DAP annually. After removal of pericarp and testa (seed coat), embryo was dissected in liquid nitrogen for lipid analysis and RNA extraction. we added in this revised manuscript.

### Lipid analysis

Total lipids were extracted from samples of freeze-dried powder using petroleum ether as solvent at 50 °C for 8 h. Analyses of fatty acid methyl esters (FAME) were performed according to the ISO,method 5509, and gas chromatography analyses of FAME were carried out using SHIMADZU GC-2014C apparatus. For each tissue and each stage of development, lipid analysis was performed in biological triplicate under a completely random experimental design. The significance of difference between main fatty acid compositions at different stages each year was tested by analysis of variance and multiple comparisons (Duncan’s new multiple range test).

### RNA extraction, mRNA purification and cDNA library construction

Total RNA was isolated from embryo using the RNeasy mini kit (QIAGEN, Germantown, MD, USA) with an additional DNase I (QIAGEN) digestion step to remove any genomic DNA contamination. The concentration of the purified RNA was determined by a Qubit2.0 fluorometer (Invitrogen, Carlsbad, CA, USA). RNA integrity was assessed by the Agilent Technologies 2100 Bioanalyzer.

According to the embryo dimension and its oil content, five annual developmental stages, i.e., early cotyledon stage, midcotyledon stage, late cotyledon stage, full cotyledon stage, and maturation stage, were designated as S1-S5 in 2012 and T1-T5 in 2013 respectively, and chosen for performing the RNA-seq analysis. The mRNA was purified from 1 μg of total RNA for RNA-Seq. cDNA library was prepared using the TruSeq RNA Sample Prep Kit (Illumina, San Diego, CA) according to the manufacturer’s instructions after mRNA purified and fragmented. The samples were then clustered and sequenced on Illumina HiSeq 2500. Deep sequencing was performed on each treatment for a 100 cycle pair end run.

### RNA-Seq data analysis and subcellular localization

RNA-Seq reads were assessed for quality control with FastQC (version 0.10.1; Babraham Bioinformatics, Cambridge, UK). All reads were assembled by trinity (r2013-02-25) with default parameters. The consistency analysis was implemented by principal component analysis (PCA). The gene transcriptional levels in PCA plots were normalized using log10 of FPKM from 10 datasets in two years.

The transcript abundances were measured as FPKM calculating by RSEM [49]. Cuffdiff 2 manuscript [50] was then used to determine levels of gene and isoform differential expression (FDR ≤ 0.05). Gene Ontology and Kyoto Encyclopedia of Genes and Genomes (KEGG) pathways were determined to be over-represented using the Fisher exact test with a false discovery rate (FDR) correction (FDR ≤ 0.05).

All the core lipid synthesis genes were conducted through subcellular localization. The subcellular localization was mutual verified by ProtComp v9.0), SignalP 4.1 Server and loctree 3. Only the gene repeats with correct locations were recruited for further research.

#### Estimation of differential gene expression

Genes were estimated to be significantly differentially expressed if expression values showed a >2-fold change with an FDR-adjusted P value ≤ 0.05 between adjoining stages for two years at least two time points, and moreover their FPKM values at either of the conditions were >10. The 5606 differentially expressed genes were grouped into 9 clusters based on their temporal expression patterns by the k-means clustering using MeV4.9 with Euclidean distance.

### Construction of co-expression network in hickory

A total of 29 lipid synthesis genes were used as the data sets to construct gene co-expression network. Instead of constructing a network based on the whole data sets, it was simply considered that the genes co-expressed with lipid-related genes as a more robust approach to survey the gene regulatory relationship over the embryo development which made further efforts help us to detect validated genes involving in lipid synthesis and temperature-inducing.

To quantify the similarity of the gene transcript abundance profiles, Pearson’s correlation coefficients (PCC) of each gene pair, was calculated following the formulas of the online help page (http://atted.jp/help/coex_cal.shtml). The genes having the PCC ≥ 0.7 with the lipid-related genes were selected to generate the co-expression networks. Using line width to indicate the correlation strength between 2 genes, red or blue line to indicate the positive or negative correlation of them, respectively.

## Availability of supporting data

The transcriptome data sets supporting the results of this article are available in the NCBI Sequence Read Archive. Accession numbers of raw data and assembled data for the ten transcriptome data are SRR2006622-SRR2006631 and GDAX00000000, respectively.

## Additional files

Additional file 1:
**Gene transcription in lipid synthesis.** (XLSX 55 kb) Additional file 2:
**Potential genes related with lipid synthesis via co-expression network.** (XLSX 24 kb)

## Abbreviations

ACCase: Acetyl-CoA carboxylase
DAG: Diacylglycerol
DAP: Day after pollination
DGAT: Diacylglycerol acyltransferase
EAR: Enoyl-ACP reductase
ER: Endoplasmic reticulum
FAD: ω-3 fatty acid desaturase
FAME: Fatty acid methyl esters
FAS: Fatty acid synthase
FATA: Acyl-ACP thioesterases A
FATB: Acyl-ACP thioesterases B
FDR: False discovery rate
FPKM: Fragments per kilobase of exon per million fragments mapped
G1,6P: Glucose 1,6-bisphosphate
G3P: Glycerol-3-phosphate
G6P: Glucose-6-phosphate
GPAT: Glycerol-3-phosphate acyl-transferase
GPDH: Glyceraldehyde-3-phosphate dehydrogenase
HAD: Hydroxyacyl-ACP dehydratase
HSP: Heat shock protein
HXK: Hexokinase
KAR: 3-ketoacyl-ACP reductase
KAS: Ketoacyl synthase
KEGG: Kyoto Encyclopedia of Genes and Genomes
LACS: Long-chain acyl-CoA synthetase
LEC1: LEAFY COTYLEDON 1
LPAT: Lysophosphatidic acid acyl transferase
LPCAT: Lysophoshatidylcholine acyltransferase
MUFAs: Monounsaturated fatty acids
PAP: Phosphatidic acid phosphatase
PC: Phosphatidylcholine
PCC: Pearson correlation coefficient
PDAT: Phospholipid:diacylglycerol acyltransferase
PDHC: Pyruvate dehydrogenase complex
PEP: Phosphoenolpyruvate
PFK: Phosphofructokinase
PKP: Phosphoenolpyruvate
PUFAs: Polyunsaturated fatty acids
PXA1: PEROXISOMAL ABC-TRANSPORTER1
SAD: Delta-9-stearoyl-ACP desaturase
SFAs: Saturated fatty acids
SDP1: SUGAR-DEPENDENT1
TAG: Triacylglycerol
WRI1: WRINKLED1
